# Roles of Peroxinectin in PGE_2_-Mediated Cellular Immunity in *Spodoptera exigua*

**DOI:** 10.1371/journal.pone.0105717

**Published:** 2014-09-05

**Authors:** Jiyeong Park, David Stanley, Yonggyun Kim

**Affiliations:** 1 Department of Bioresource Sciences, Andong National University, Andong, South Korea; 2 Biological Control of Insects Research Laboratory, USDA/Agricultural Research Service, Columbia, Missouri, United States of America; Natural Resources Canada, Canada

## Abstract

**Background:**

Prostaglandins (PGs) mediate insect immune responses to infections and invasions. Although the presence of PGs has been confirmed in several insect species, their biosynthesis in insects remains a conundrum because orthologs of the mammalian cyclooxygenases (COXs) have not been found in the known insect genomes. PG-mediated immune reactions have been documented in the beet armyworm, *Spodoptera exigua*. The purpose of this research is to identify the source of PGs in *S. exigua*.

**Principal Findings:**

Peroxidases (POXs) are a sister group of COX genes. Ten putative POXs (*SePOX-A ∼ SePOX-J*) were expressed in *S. exigua*. Expressions of *SePOX-F* and *-H* were induced by bacterial challenge and expressed in the hemocytes and the fat body. RNAi of each *POX* was performed by hemocoelic injection of their specific double-stranded RNAs. dsPOX-F or, separately, dsPOX-H, but not the other eight dsRNA constructs, specifically suppressed hemocyte-spreading behavior and nodule formation; these two reactions were also inhibited by aspirin, a COX inhibitor. PGE_2_, but not arachidonic acid, treatment rescued the immunosuppression. Sequence analysis indicated that both POX genes were clustered with *peroxinectin* (*Pxt*) and their cognate proteins shared some conserved domains corresponding to the Pxt of *Drosophila melanogaster*.

**Conclusions:**

*SePOX-F* and *-H* are Pxt-like genes associated with PG biosynthesis in *S. exigua*.

## Introduction

Insect innate immunity is composed of cellular and humoral immune responses [1]. Cellular immune responses are performed by hemocytes and include phagocytosis, nodulation, and encapsulation; these reactions begin immediately upon microbial infection [2]. Humoral immune responses begin about 6–12 h post-infection (PI) and they include production of antimicrobial peptides (AMPs) and plasma melanization [3]. Pattern recognition receptors perceive the presence of invaders and activate specific immune responses via immune mediators [4]. Depending on which recognition molecules are activated, Toll and/or IMD signal pathways are triggered to signal intracellular expression of specific AMPs [5]. The recognition signal also launches melanization responses by initiating a prophenoloxidase (PPO) activation cascade [6]. Immune mediators, including cytokines, biogenic monoamines, and various eicosanoids, particularly PGs, mediate and coordinate cellular immune responses [7]. PGs act in cross-talk between intracellular immune signals [8].

Eicosanoids are a group of C20 polyunsaturated fatty acids mostly derived from arachidonic acid (AA) [9]. AA is released from biomembrane phospholipids (PLs) by catalytic activity of phospholipase A_2_ (PLA_2_) [10], [11]. In the mammalian model, the free AA is oxygenated by cyclooxygenases (COXs) to form PGs, by lipoxygenases (LOXs) to form hydroxyeicosanoids and leukotrienes (LTs) or by epoxygenases to form epoxytrienoic acids [12]. Eicosanoids mediate insect cellular and humoral immune responses to various pathogens including bacteria, fungi, endoparasitoid nematodes, eggs of parasitoid wasps, and viruses in insects [9], [12]. PGs and LTs mediate the hemocyte nodulation reaction to bacterial challenge [13]. PGs, but not LTs, mediate microaggregation [14]. The release of PPO from circulating oenocytoids (a class of hemocytes) into hemolymph is mediated solely by PGs in *Spodoptera exigua* [15]. PGs act in insect homeostatic physiology beyond immunity. In reproduction, PGs act in follicle development from vitellogenesis to choriogenesis in *Bombyx mori* [16] and, in follicle development and in the temporal sequence of expressing genes encoding egg-shell proteins in *Drosophila* [17], [18]. In the cricket, *Teleogryllus commodus*, PGE_2_ triggers egg-laying behavior of virgin females, mimicking a mating effect [19]. PGs, but not LOX products, mediate secretory activity of Malpighian tubules of *Aedes aegypti* [20] and *Formica polyctena* [21]. In rectum, PGE_2_ exhibits a dose-dependent stimulation of fluid reabsorption in *Locusta migratoria* [22]. PGs also influence gene expression in an established insect cell line [23]. We infer that PGs mediate a wide range of physiological processes in insects many of which remain to be identified. Eicosanoids, generally, have been recorded, and shown to exert physiological actions, in all invertebrates that have been studied in this regard [12], [24].

Various PGs have been identified in insects [9]. PGE_2_ was identified in the principal, but not stellate, cells of Malpighian tubules of *A. aegypti* by immunohistochemical staining [20]. PGF_2_α was identified in hemolymph of *Pseudaletia unipuncta* by fluorescence-HPLC and confirmed by mass spectrometry [25]. *In vitro* preparations of the *Manduca sexta* midgut produced five PGs, PGA, PGB_2_, PGD_2_, PGE_2_, and PGF_2_α [26]. The precursor for PG biosynthesis, AA, is mainly associated with cellular PLs. Eicosanoid biosynthesis begins with release of AA from PLs by PLA_2_s, which have been identified in *Drosophila* genome [27], [28]. Four immune-associated PLA_2_s are expressed in *Tribolium castaneum* [29]. However, there is no ortholog of mammalian COXs in the annotated genomes of *D. melanogaster*, *A. aegypti, Anopheles gambiae, Apis mellifera, B. mori* or *T. castaneum* [30], which drives the question of how can the presence and actions of PGs in insect tissues be understood in these insects lacking a COX? The question can be resolved by identifying an alternative PG biosynthetic pathway.

In *Drosophila*, PG signaling is required for follicle development. The requisite PGs are produced via a specific peroxidase (POX) classified as a peroxinectin (Pxt). Mutant flies lacking this gene function are sterile, however, follicle development can be restored by heterologous expression of a vertebrate COX gene in the mutants [17]. Tootle and Spradling [18] conclude that the *Drosophila Pxt* is responsible for PG biosynthesis. The idea of an alternative mechanism of PG biosynthesis prompted our hypothesis that genes encoding one or more POXs are responsible for PG production in *S. exigua*. We tested our hypothesis by identifying ten SePOX genes from transcriptomes of *S. exigua*. In the paper we report that two of the ten *SePOX*s encode enzymes that produce immune-mediating PGs.

## Results

### Classification of ten *SePOX*s

Interrogation of two *S. exigua* transcriptomes (PRJNA192625 and Spodobase (http://bioweb.ensam.inra.fr/spodobase/)) yielded ten *SePOXs* (Fig. S1, GenBank accession numbers: KJ995802–KJ995811). The predicted amino acid sequences were compared with sequences of other POX-related genes from vertebrates and invertebrates (Table 1). This sequence analysis showed three clusters of Pxt/COX, POX, and peroxiredoxin (PRX) subfamilies, where ten *SePOX*s were separately clustered: six genes in Pxt/COX, three in PRX, and one in POX.

**Table 1:** Peroxidases (SePOXs) collected from transcriptomes^1^ of *Spodoptera exigua*.

Group	Genes	Accession number of GenBank	ORF (bp)	MW (kDa)	Blast	
					Gene^2^ (Species^3^)	E-value
POX	SePOX-A	KJ995802	399	14.3	GSH-POX (Bm)	2e-12
	SePOX-B	KJ995803	312	11.8	GSH-POX (Bm)	5e-42
	SePOX-E	KJ995806	723	27.7	POX (Dp)	8e-128
	SePOX-G	KJ995808	1278	42.7	POX (Bm)	4e-177
	SePOX-I	KJ995810	>960	-	POX (Tc)	2e-144
	SePOX-J	KJ995811	1833	69.2	POX (Bm)	0.0
PRX	SePOX-C	KJ995804	588	22.0	PRX (Ha)	2e-138
	SePOX-D	KJ995805	>480	-	PRX (Px)	3e-102
COX/Pxt	SePOX-F	KJ995807	2067	76.1	Pxt (Pl)	3e-120
	SePOX-H	KJ995809	2262	84.3	Pxt (Bm)	0.0

1 Two transcriptomes of NCBI GenBank with accession number of PRJNA192625 and Spodobase (http://bioweb.ensam.inra.fr/spodobase/)

2 ‘POX’, ‘Pxt’, and ‘PRX’ represent peroxidase, peroxinectin, and peroxiredoxin, respectively.

3 Species include *Bombyx mori* (Bm), *Danaus plexippus* (Dp), *Helicoverpa armigera* (Ha), *Pacifastacus leniusculus* (Pl), *Plutella xylostella* (Px), and *Tribolium castaneum* (Tc).

### Expression patterns of ten *SePOX*s

**Figure 1:**
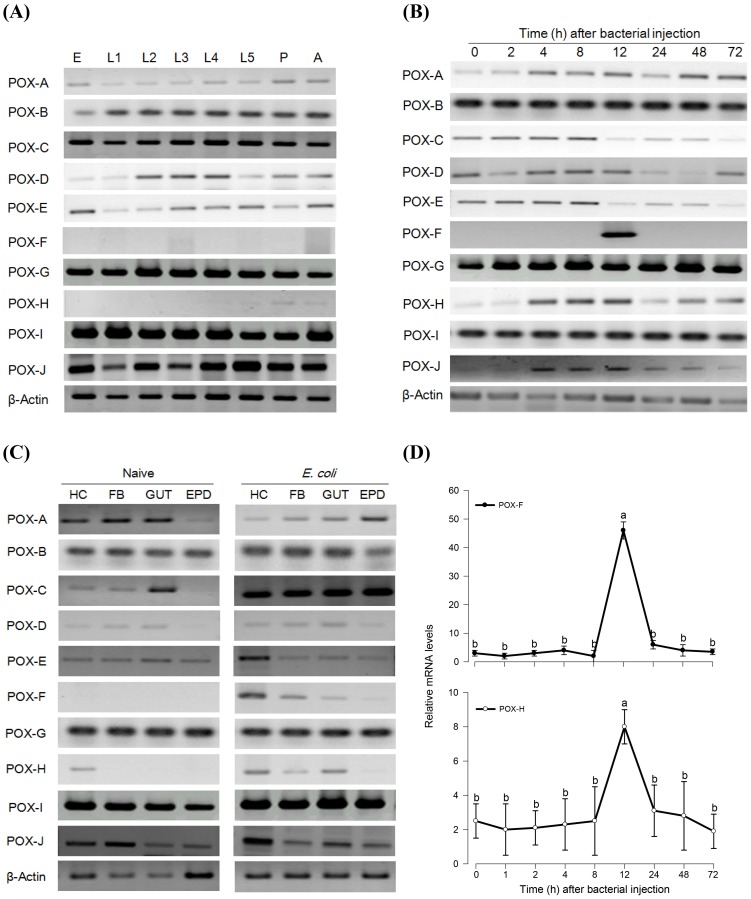
Expression patterns of ten peroxidases (POXs) of *Spodoptera exigua* analyzed by RT-PCR. (A) Expressions in different developmental stages: ‘E’ for egg, ‘L1-L5’ for first to fifth instar larvae, ‘P’ for pupa, and ‘A’ for adult. (B) Expressions after bacterial challenge to L5 larvae, injected with 5×10^5^ cells of *Escherichia coli*. (C) Expressions in indicated tissues of L5 larvae: ‘HC’ for hemocytes, ‘FB’ for fat body, ‘GUT’ for midgut, and ‘EPD’ for epidermis. L5 was challenged with *E. coli* as described above and incubated for 12 h. (D) qPCR analysis of two POX genes after the bacterial challenge as described above. Three independent replications were performed to measure means and standards of their expressions. Different letters above error bars indicate significantly among means of Type I error  = 0.05 (LSD test).

During the entire developmental stages from egg to adult, most *SePOX*s except *SePOX-F* and *-H* were constitutively transcribed (Fig. 1A). Without bacterial challenge, *SePOX-F* transcription was not detected and *SePOX-H* was transcribed at a low, constitutive level. However, following bacterial challenge, transcription of both genes was remarkably increased, *POX-F* at 12 PI and *POX-H* from 4–12 h PI (Fig. 1B). Bacterial challenge did not influence expression of the other *SePOX*s. The inducible expression of *SePOX-F* and *-H* was analyzed in tissues of bacterial-challenged larvae (Fig. 1C). These genes were expressed in the hemocytes and the fat body at 12 h PI for *SePOX-F* and 4–72 h PI for *SePOX-H*. Levels of gene induction were assessed by qPCR (Fig. 1D). *SePOX-F* transcript levels increased by 48-fold, and *SePOX-H* by 8-fold, both between 8 and 24 h PI.

### Influence of dsRNA treatments on gene expression and hemocyte behavior

**Figure 2:**
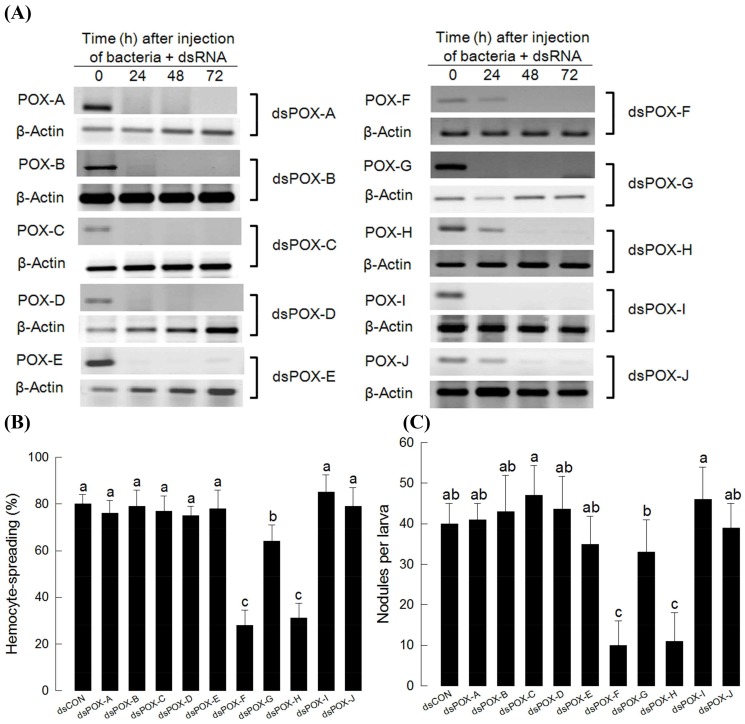
RNA interference (RNAi) of ten peroxidases (POXs) of *Spodoptera exigua* and functional assay with respect to cellular immune responses. (A) RNAi using double-stranded RNAs (‘dsPOXs’) specific to each of the ten POXs. To induce POX genes, 5×10^5^ *Escherichia coli* cells were injected to L5 larvae along with dsRNA (500 ng per larva). (B) Hemocyte-spreading assay after dsRNA treatment. At 48 h after dsRNA injection, hemocytes were collected for bioassay. (C) Nodulation assay. At 48 h after dsRNA treatment, 5×10^5^ cells of *E. coli* were injected and incubated for 8 h at 25°C. Control dsRNA was prepared against a viral gene, CpBV-ORF302. Each treatment was independently replicated three times. Different letters above error bars indicate significant difference among means at Type I error  = 0.05 (LSD test).

Gene-specific dsRNA treatments inhibited expression of all ten *SePOX*s for at least 72 h (Fig. 2A). The dsRNA treatments directed to *SePOX-F* or, separately, *SePOX-H*, but not to the other *SePOX*s, effectively inhibited the hemocyte spreading behavior (Fig. 2B) and nodulation (Fig. 2C).

### PGE_2_ rescues RNAi-induced immunosuppression

**Figure 3:**
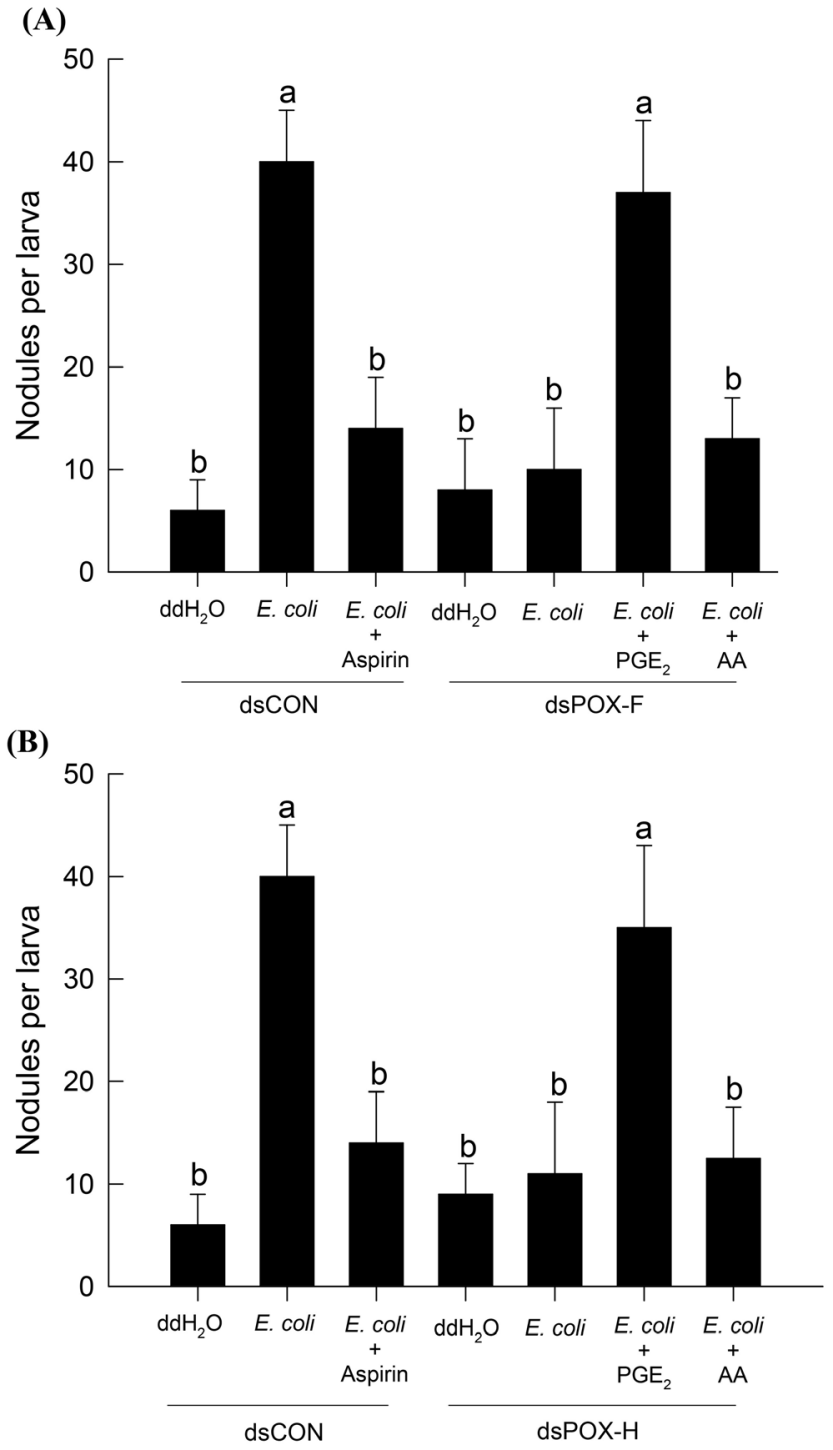
Injection of PGE_2_ reversed the immunosuppression induced by RNA interference (RNAi) of POX-F (A) and POX-H (B). Immunosuppression was recorded as decreased nodule formation induced by injection of dsRNA, in which ‘dsPOX-F’ specific to POX-F or dsPOX-H specific to POX-H, was injected in a dose of 500 ng per larva and subsequently incubated for 48 h at 25°C. For nodulation, 5×10^5^ cells of *E. coli* were injected to each test larva and incubated for 8 h at 25°C. Aspirin, a COX-specific inhibitor, was injected in a dose of 100 ng per larva along with the bacterial challenge. Control dsRNA (‘dsCON’) was prepared against a viral gene, CpBV-ORF302. Each treatment was independently replicated three times. Different letters above error bars indicate significant difference among means at Type I error  = 0.05 (LSD test).

Our results with hemocyte-spreading and nodulation reactions indicate that SePOX-F and SePOX-H are necessary to evoke cellular immune responses. As seen in our previous reports [8], [31], PGs mediate hemocyte-spreading behavior and nodulation. Aspirin is a specific inhibitor of mammalian and invertebrate forms of COX [32]. Our data show that aspirin treatments significantly suppressed hemocyte nodule formation (Fig. 3A, B). Inhibition of nodule formation by silencing *SePOX-F* or, separately, *-H*, was rescued by the addition of PGE_2_., but not by AA.

### Pxt-like structures of *SePOX-F* and *SePOX-H*

**Figure 4:**
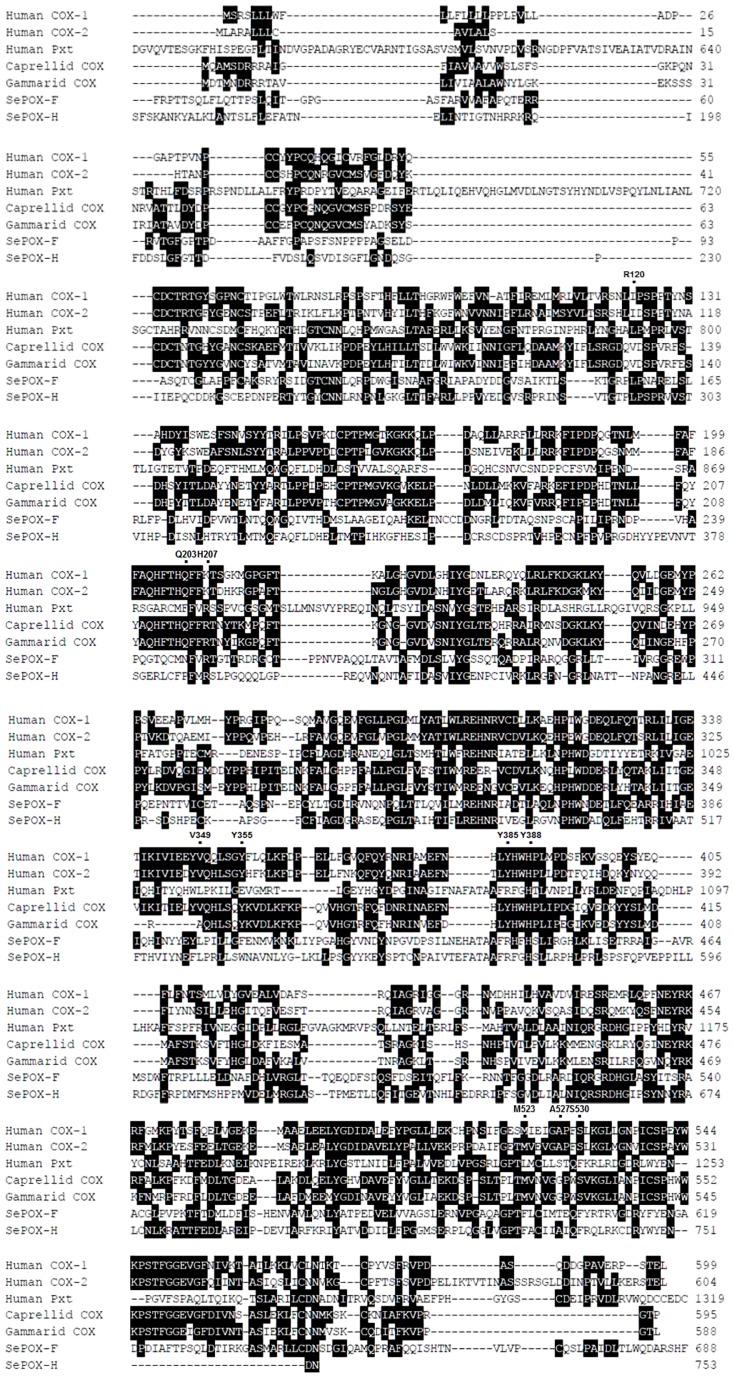
Identification of *S. exigua* POX-F and POX-H as Pxt-like genes. An alignment of POX-F and POX-H with other COX/Pxt genes: Hs COX-1 (*Homo sapiens* COX1, P23219), Hs COX-2 (*Homo sapiens* COX2, P35354), Hs Pxt (*Homo sapiens* Pxt, NP036425), Caprellid COX (*Caprellid spp*. COX, GQ190795), and Gammarid COX (*Gammarid spp*. COX, GX180796). Dot spots indicate residues conserved in COX.

*SePOX-F* and *-H* cluster with COX/Pxt genes (Table 1). To clarify the similarity with COX in terms of catalytic sites, these two POXs were aligned with vertebrate and invertebrate COX genes (Fig. 4). Conserved residues in COX active sites occur in crustacean COX genes especially at Arg 120, Gln 203, His 207, Tyr 355, Tyr 385, His 388, Met 523, and Ser 530. However, these sites do not occur in either SePOX-F, or SePOX-H except Gln 203 and His 207.

**Figure 5:**
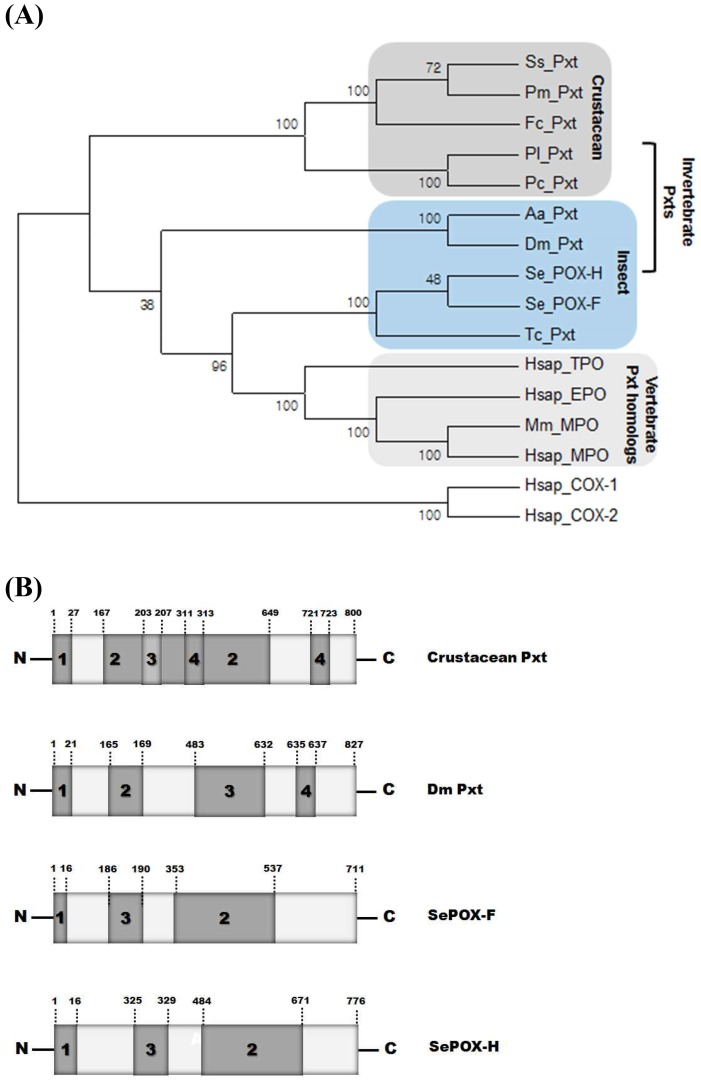
Identification of *S. exigua* POX-F and POX-H as Pxt-like genes. (A) A phylogenetic analysis of Pxt genes from invertebrates and vertebrates using maximum likelihood. Number of bootstrap replication is 1,500 and substitutions type is amino acid. ‘POX’, ‘Pxt’, ‘TPO’, ‘MPO’, and ‘EPO’ represent peroxidase, peroxinectin, thyroid peroxidase, myeloperoxidase, and eosinophil peroxidase, respectively. Sequence were retrieved from GenBank: Pm Pxt (*Penaeus monodon*, AF188840), Ss Pxt (*Scylla serata*, ACF32960), Fc Pxt (*Fenneropenaeus chinensis*, DQ172834), Pc Pxt (*Procambarus clarkia*, ADW79421), Pl Pxt (*Pacifastacus lenusculus*, X91409), Dm Pxt (*Drosophila melanogaster*, NP650648), Aa Pxt (*Aedes agepyti*, XP001657082), Tc Pxt (*Tribolium castaneum*, XP973386), Hsap TPO (*Homo sapiens*, NM175721), Hsap EPO (*Homo sapiens*, NM000502), Hsap MPO (*Homo sapiens*, BC130476), Hsap COX-1 (*Homo sapiens* COX1, P23219), Hsap COX-2 (*Homo sapiens* COX2, P35354), and Mm MPO (*Mus musculus*, AY560847). (B) Comparison of conserved domains of SePOX-F and SePOX-H with those of a crustacean Pxt (*Pacifastacus lenusculus*, X91409) and Dm Pxt. 1; signal peptide, 2; substrate binding site, 3; POX activity site, 4; integrin binding site.

*SePOX-F* and *-H* were aligned with *Pxt* genes of invertebrates, *Pxt* homologs of vertebrates and *COX* genes of vertebrates (Fig. 5A). The *Pxt* genes form three clusters, insect *Pxt*s, crustacean *Pxt*s, and vertebrate *Pxt* homologs. Among Pxts, the crustacean type has a wide substrate-binding domain containing a core catalytic site, while the insect type has a narrow substrate-binding domain distinct from a core catalytic site (Fig. 5B). Among insect *Pxt* genes, *SePOX-F* and *-H* lack the *D. melanogaste*r Pxt integrin binding site, Arg-Gly-Asp.

## Discussion

The data reported in this paper strongly support our hypothesis that genes encoding SePOX-F and -H are responsible for PG production in *S. exigua*. Several points make up the central argument. First, the COX/Pxt genes in this study cluster as a separate group. Second, of the ten *SePOX*s we analyzed, expression of *SePOX-F* and *-H*, but not the other eight SePOX genes, were induced by bacterial challenge. Third, gene-silencing dsRNA constructs specific to each of the ten *SePOX*s effectively inhibited expression of all ten genes. In separate experiments, dsPOX-F and dsPOX-H, but none of the other eight dsRNA constructs, effectively disabled both immune functions. Fourth, the inhibitory influence of dsPOX-F and dsPOX-H treatments on nodulation was effectively reversed by treating dsRNA-injected larvae with PGE_2_, but not with AA. Fifth, while *SePOX-F* and *SePOX-H* do not share the catalytic amino acids known in mammalian COX genes, their catalytic sites are shared with the *D. melanogaster* Pxt, which also produces PGs. Taken together, these five points form a very strong line of reasoning supporting our view that *SePOX-F* and *-H* act in the biosynthesis of immune-mediating PGs.

Although the biological significance of PGs and other eicosanoids in insect biology is solidly established [9], [12], there is very little knowledge about how insects produce PGs. This may come as surprising, given the several papers characterizing PG biosynthesis in insect issues, cited in [9]. Yet, for several reasons the received orthodoxy informs that PLA_2_, COX, and LOX are the three main pillars of insect eicosanoid biosynthesis, as known from the mammalian model. First, insect PLA_2_ activity that favors arachidonyl-containing PL substrate has been described in *M. sexta* hemocytes [33]. More recently, Kim and his colleagues identified four PLA_2_–encoding genes that act in eicosanoid-mediated immune responses in *T. castaneum* [29]. These findings solidly support the first pillar of eicosanoid biosynthesis and lend credence to the idea that COX and LOX would also act in insect eicosanoid biosynthesis. Second, specific pharmaceutical inhibitors of each of these enzymes inhibit eicosanoid biosynthesis and they inhibit targeted physiological processes, such as cellular immune functions and primary urine formation in Malpighian tubules [9], [12]. Third, the biochemistry of PG biosynthesis has been described in considerable detail in tissues prepared from the tobacco hornworm, *M. sexta* and other insect species. The biochemically detailed studies are consistent with PG biosynthesis via a COX. For example, PG biosynthesis is inhibited in *in vitro* reactions conducted in the presence of non-steroidal anti-inflammatory drugs (NSAIDs), which inhibit mammalian COXs and the enzyme co-factor requirements appear to be similar in mammalian and insect preparations [9], [12]. It follows that the (admittedly limited) literature on insect PG biosynthesis conveys the view that the biochemical mechanisms are similar to the mammalian model.

Although genes encoding COXs have not been reported for insects, a COX has been described for the human body louse, *Pediculus humanus*; this COX is much smaller than other COX-encoding genes and phylogenetic analysis indicates it lies separate from known COX genes [30]. This leaves the possibility that some insects may express a vertebrate-like COX. Relative to an alternate PG-producing mechanism, the actions of *SePOX-F* and *-H*, are required for PG-mediated cellular immune functions in *S. exigua*. This is apparent in our end-product rescue experiments. Treating larvae with dsPOX-F effectively inhibited nodulation, which was strongly reversed by PGE_2_. SePOX-H was fully operational in cells exposed to dsPOX-F, but the catalytic operations of SePOX-H alone did not yield an immune-mediating product. The same outcome holds for silencing *SePOX-H*, which would leave *SePOX-F* operational. This finding opens a question that lies beyond the scope of this paper: do these two genes encode proteins with different catalytic functions, each of which is required for PG biosynthesis, or is the summed product of both genes necessary to produce a physiologically relevant amount of PG?

Aspirin is the archetypal NSAID with a long history. The acetyl group of aspirin forms a covalent bond with a serine residue in vertebrate COX, thereby permanently inhibiting PG biosynthesis until more COX protein is synthesized. Aspirin effectively blocked the mediating actions of SePOX-F and SePOX-H, which was reversed upon addition of PGE_2_, but not by AA. AA did not rescue the immunosuppressive effects of dsPOX because PGs, not AA, are the end-products of SePOX-F and –H catalysis. We infer from the rescuing effect of exogenous PGE_2_ these two *SePOX*s are associated with PG biosynthesis. Sequence analysis of *SePOX-F* and *-H* indicates they are clustered with the COX/Pxt group. Although these two SePOX genes do not have the COX active site expected based on the mammalian model background (e.g., Arg 120), they retain the Pxt active site domain containing four heme-binding α-helical domains. Based on these results we classify the two SePOX proteins as Pxt-like and conclude they act in PG biosynthesis.

Pxt was first identified as a cell adhesion molecule in a crayfish due to its POX catalytic domain and integrin-binding motif (KGD: Lys-Gly-Asp) [34]. Among crustacean species, Pxt also mediates hemocyte degranulation [34], immobilization of microbial pathogens, phagocytosis, encapsulation, nodule formation [35], [36], opsonization [37], and a humoral immune response [35]. Pxt is homologous to a vertebrate myeloperoxidase, but does not occur in vertebrates [38]. Our phylogenetic analysis indicates to us that the insect Pxts cluster separately from crustacean Pxts and that *SePOX-F* and *SePOX-H* cluster with the other insect *Pxt*s. This phylogenetic analysis is supported by a previous analysis by Vizzini et al. [38], who suggest that insect Pxts may not behave like their crustacean counterparts. Although the idea has not been tested, we speculate that some of these crustacean Pxts also act in PG biosynthesis.

The first insect PGs were identified by radioimmunoassay in the house cricket, *Acheta domesticus*, in which male crickets synthesized PGE_2_ in reproductive tracts and PGE_1_ in spermatophores [39]. Another cricket, *T. commodus*, also produces PGE_2_ and PGF_2α_ in the spermathecae [40]. Murtaugh and Denlinger [41] assessed these two PGs in six different species and tissues within species, finding substantial variation in their amounts. In their work with houseflies, *Musca domestica*, Wakayama et al. [42] determined the subcellular localization of PG synthesis in the microsomal fraction of whole-animal homogenates. In a lepidopteran species, *M. sexta*, the microsomal fractions of the fat body and the hemocytes synthesized PGA_2_, PGE_2_, PGD_2_, and PGF_2α_ [43], [44]. Bacteria-challenged true armyworms synthesized and released PGF_2α_ into the plasma [25]. The authenticity of PG identifications have been confirmed by obtaining mass spectra of the compounds [9], [12]. Thus, insects certainly produce physiologically active PGs. Those insects lacking COX genes synthesize PGs by at least one alternative biosynthetic pathway involving Pxt.

## Materials and Methods

### Insect rearing and bacterial culture

Larvae of *S. exigua* were collected from welsh onion and reared on an artificial diet [45] at 25°C, 16∶8 (L∶D) h photoperiod, and RH 60±5%. For bacterial challenge, *Escherichia coli* Top10 (Invitrogen, Carlsbad, CA, USA) was cultured overnight in Luria-Bertani medium (Difco, Sparks, MD, USA) at 37°C in a shaking incubator at 270 rpm.

### Chemicals

PGE_2_ [5Z,11α,13E,15S]-11,15-dihydroxy-9-oxoprosta-5,13-dienoic acid, arachidonic acid [5,8,11,14-eicosatetraenoic acid] and aspirin [2-acetoxybenzoic acid] were purchased from Sigma-Aldrich Korea (Seoul, Korea). Anticoagulant buffer (ACB) was prepared with 186 mM NaCl, 17 mM Na_2_EDTA, and 41 mM citric acid. The ACB was adjusted to pH 8.0 by addition of NaOH.

### RNA extraction

Total RNA was extracted using the Trizol reagent (MRC, Cincinnati, OH, USA) according to manufacturer's instructions. RNA was extracted from selected developmental stages, times after bacterial injection, and tissues of three day old fifth instar larvae (L5D3). Tissues analyzed in this study included hemocytes (HC), fat body (FB), gut (GUT) and epidermis (EPD), which were isolated from L5D3. The extracted RNAs were treated with RNase-free DNase (TaKaRa, Shinga, Japan). RNA quality was assessed on agarose gels and quantities were determined on a spectrophotometer.

### RT-PCR

After the absence of DNA contamination was confirmed by PCR with the RNA template, the first strand cDNA was synthesized from the RNA extract (1 µg per reaction) by reverse transcription using RT-premix (Intron Biotechnology, Seoul, Korea) containing an oligo dT primer (5′- CCAGTGAGCAGAGTGCGAGGACTCGAGCTCAAGCTTTTTTTTTTTTTTTTT-3′). Ten SePOX genes (*SePOX-A∼SePOX-J*) were analyzed by RT-PCR using the cDNAs. All PCRs used 40 amplification cycles under 94°C denaturation for 30 sec, gene-specific annealing temperatures for 30 sec and 72°C extension for 30 sec using Taq polymerase (GeneAll, Seoul, Korea) with gene-specific primers (Table 2).

**Table 2:** Primers used in RT-PCR in this study.

Genes	Sequences	Annealing (°C)
β-actin	TGGCACCACACCTTCTAC	50
	CATGATCTGGGTCATCTTCT	
POX-A	CCAAGCTTGTAGCGCCCATAG	50
	GCCCAATGTACCTCCTTGC	
POX-B	CTGCTGAAACTGTATCAGCC	49
	CATTGACCGCGACCTTCTC	
POX-C	CCCATTTCAACAAGATGCCTC	50
	GTCGACGAAGTATTCCTGG	
POX-D	CTTGATCGTGGCTGCGTTTG	49
	TCAGTGACAGGGCAAAGGAG	
POX-E	GGTAGCAGATATCCTAAGCG	50
	GTATCGGTCAGATATCCGAG	
POX-F	CGACAACATCGCAACTGTTC	50
	TGGTTCACACGAATGTCACC	
POX-G	CAGCCCTACGTGAAAGAACC	47
	AGAAGTATCTGTCTCCTGCC	
POX-H	CGTCTAGAACTTTCTGCGTG	50
	GACCAGGCAAACTTCTCATG	
POX-I	GGCAGGATATCCTGTCATTG	55
	CGTGCAGTCTTCCATGGTTAG	
POX-J	GACCGCTTCTCAAGTTGCTG	55
	TTGTGTCACGAGTGTTGGTG	
T7 POX-A	TAATACGACTCACTATAGGGAGACCAAGCTTGTAGCGCCCATAG	50
	TAATACGACTCACTATAGGGAGAGCCCAATGTACCTCCTTGC	
T7 POX-B	TAATACGACTCACTATAGGGAGACTGCTGAAACTGTATCAGCC	49
	TAATACGACTCACTATAGGGAGA CATTGACCGCGACCTTCTC	
T7 POX-C	TAATACGACTCACTATAGGGAGACCCATTTCAACAAGATGCCTC	50
	TAATACGACTCACTATAGGGAGAGTCGACGAAGTATTCCTGG	
T7 POX-D	TAATACGACTCACTATAGGGAGACTTGATCGTGGCTGCGTTTG	49
	TAATACGACTCACTATAGGGAGATCAGTGACAGGGCAAAGGAG	
T7 POX-E	TAATACGACTCACTATAGGGAGAGGTAGCAGATATCCTAAGCG	50
	TAATACGACTCACTATAGGGAGAGTATCGGTCAGATATCCGAG	
T7 POX-F	TAATACGACTCACTATAGGGAGACGACAACATCGCAACTGTTC	50
	TAATACGACTCACTATAGGGAGATGGTTCACACGAATGTCACC	
T7 POX-G	TAATACGACTCACTATAGGGAGACAGCCCTACGTGAAAGAACC	47
	TAATACGACTCACTATAGGGAGAAGAAGTATCTGTCTCCTGCC	
T7 POX-H	TAATACGACTCACTATAGGGAGACGTCTAGAACTTTCTGCGTG	50
	TAATACGACTCACTATAGGGAGAGACCAGGCAAACTTCTCATG	
T7 POX-I	TAATACGACTCACTATAGGGAGAGGCAGGATATCCTGTCATTG	55
	TAATACGACTCACTATAGGGAGACGTGCAGTCTTCCATGGTTAG	
T7 POX-J	TAATACGACTCACTATAGGGAGAGACCGCTTCTCAAGTTGCTG	55
	TAATACGACTCACTATAGGGAGATTGTGTCACGAGTGTTGGTG	

### Quantitative PCR

Quantitative PCR (qPCR) used SYBR Green Realtime PCR master mix (Toyobo, Osaka, Japan) in a 7500 real time PCR system according to the manufacturer's instructions. The reaction volume was 20 µL including each 10 µM of forward and reverse primers, and 90 ng of template cDNA. After activation of Hot-start Taq DNA polymerase at 94°C for 15 min, the reaction condition included 35 cycles of 30 sec at 94°C, 30 sec at 50°C, and 30 sec at 72°C with final extension for 5 min at 72°C with gene-specific primers (Table 2). Fluorescence values were measured and amplification plots were generated in real time by an Exicycler TM program. Quantitative analysis followed a comparative C_T_ method [46].

### RNA interference (RNAi)

RNAi was performed with gene-specific dsRNAs, prepared using the Megascript RNAi kit according to the manufacturer's instructions (Ambion, Austin, TX, USA). Briefly, each gene fragment was produced by PCR using each pair of the gene-specific primers containing a T7 RNA polymerase promoter at the 5′ end (Table 2). Sense and antisense RNA strands were synthesized using T7 RNA polymerase at 37°C for 3 h. The resulting dsRNA was mixed with Metafectene PRO (Biontex, Plannegg, Germany) at a 1∶1 volume ratio and incubated at 25°C for 30 min to form liposomes. Two µL of the dsRNA (100 ng) solution was injected into the hemocoel of day old fifth instar larvae (L5D1). The microinjections were performed with a Hamilton syringe (Hamilton, Reno, Nevada, USA) equipped with a 26 gauge needle. Knock-down efficacy of RNAi was assessed by RT-PCR of each gene at selected times PI. For control dsRNA, dsRNA specific to a viral gene, CpBV-ORF302, was prepared and similarly injected [47].

### Hemocyte-spreading analysis

Hemolymph was collected by cutting prologs of the treated larvae and mixed with the same volume of ACB. After centrifugation at 200×g at 4°C for 5 min, the pellet was resuspended in 1 mL of ACB and incubated for 30 min on ice. After centrifugation at 200×g for 5 min, the 700 µL supernatant was discarded and replaced with 700 µL of TC-100 insect cell culture medium (Welgene, Daegu, Korea). Bioassays were performed in 96-well culture plates (SPL, Pocheon, Korea), where each well contained 50 µL of test hemocyte sample. The plates were kept under darkness at 25°C for predetermined periods (10, 20, 30 min). Hemocyte-spreading behavior was assessed by counting the number of cells displaying cytoplasmic extension. One hundred hemocytes from a randomly selected field of view under a phase contrast microscope were assessed at 400x magnification (IX70, Olympus, Tokyo, Japan) for each replicate. Each treatment was independently replicated three times.

### Nodulation analysis

L5D3 larvae were surface-sterilized with 95% ethanol. Two µL of *E. coli* (2×10^4^ cells) were injected into the hemocoel by the micro-syringe. After 2 h at 25°C, melanized and dark nodules were counted under a microscope (SZX9, Olympus, Tokyo, Japan) at 50x magnification. Control insects were injected with 2 µL of PBS. Each treatment was independently replicated three times.

### Influence of aspirin or PGE_2_ on nodule formation

Aspirin was dissolved in PBS (at 50 µg/mL) and PGE_2_ was dissolved in ethanol (at 100 µg/mL). L5D3 larvae were co-injected with 2 µL of *E. coli* (2×10^4^ cells) and 2 µL of the test solution (4 µL injections). Nodulation was assessed at 8 h PI as described above. Each treatment was independently replicated three times.

### Data analysis

All studies were performed in three independent biological replicates and plotted by mean ± standard deviation using Sigma plot. Means were compared by a least squared difference (LSD) test of one way ANOVA using PROC GLM of SAS program [48] and discriminated at Type I error  = 0.05.

## Supporting Information

Figure S1 **cDNA sequences of ten peroxidases (** ***SePOX*** **s) of** ***Spodoptera exigua*** **(GenBank accession numbers: KJ995802–KJ995811).** Shaded boxes indicate start and stop codons.
(DOC)
