# Supplementary material for: Roles of Peroxinectin in PGE2-Mediated Cellular Immunity in Spodoptera exigua
Source: PLoS One. 2014 Sep 5;9(9):e105717. doi: 10.1371/journal.pone.0105717 (PMC4156296; doi:10.1371/journal.pone.0105717)
Supplement: Figure S1 — cDNA sequences of ten peroxidases ( SePOX s) of Spodoptera exigua (GenBank accession numbers: KJ995802–KJ995811). Shaded boxes indicate start and stop codons. (DOC) [file pone.0105717.s001.doc]

***SePOX-A***

TTTTTGTAGTGAACATGACAATCGGTTTTAGAATTTCCAAGCTTGTAGCGCCCATAGTCGGGAACGTTATT 71

MTIGFRISKLVAPIVGNVI

TGTTTATCTCGAGCCCAATTTAGTGCTGTAAAAATGAGTAATCCCGATTATAAAGCTGCGACATCGATCCA 142

CLSRAQFSAVKMSNPDYKAATSIH

TGAATTCACCGTGAAAAATATAAAGGGTGAAGATGTGAAATTGGATGTTTACAAAGGTCATGTCTGCATCA 213

EFTVKNIKGEDVKLDVYKGHVCII

TTGTGAATGTGGCCTCACAGTGCGGGCTGACTGCGAATAATTACAAGCAGTTGAATGAATTATACGAGAAA 284

VNVASQCGLTANNYKQLNELYEK

TATGCTGAGGACAAAGGTCTGCGCATCCTTGCCTTCCCCTGCAATCAGTTTGCTGGCCAGGAGCCTGGTAA 355

YAEDKGLRILAFPCNQFAGQEPGN

TCCCGAGGATATTGTTTGCTTTGCTTCTGAAAGGAAAGTGAAGTTTGACCTGTTTGAAAAAATTGATGTCA 426

PEDIVCFASERKVKFDLFEKIDVN

ACGGAGATAATGCCAGTCCCTTGTGGAAGTTCTTGAAGCACAAGCAAGGAGGTACATTGGGCAATTTCGTC 497

GDNASPLWKFLKHKQGGTLGNFV

AAATGGAACTTCACCAAATTTATTGTGGACAAGGATGGGGTACCAGTTGAGCGTCATGGACCCAATGTGGA 568

KWNFTKFIVDKDGVPVERHGPNVD

CCCATTGGACTTGGTCAAATCCCTGGAGAAGTACTGGTGATCAGCATCATGGAAAAATGGCTCTGCATTTT 639

PLDLVKSLEKYW

ATTTTTTTACGTCATAGTAAGCTTTAGTCAACTTGTAGATGGTTTTCAACAGTTTCTTCTTTCTCATCACA 710

GCATTTAACTTTTACTGTTCTTTACTCTAAATGTTTATTATACAATTTTTCACGTTTCTTAGAGGTGGCTA 781

CTCTTCGTAGCCATTTTCAAACAATTATGCACACAAAATATAATACTTATTAAGCATGTCACGGCATGTCG 852

AAAGATTATTGTTAGTTGTTAAAAACTTATAAAATATAATATATATTTATGTAAAAAAAATCTAGCAAACA 923

CATTGTGTGATATATTGAGTAGTTGTTAAGAACTATTGTTTTATTTTTTTGTGATAAAATATAT 987

***SePOX-B***

AACCTGAACCAGCCGAATCAAAAACTGCTGAAACTGTATCAGCCAAGAAGTCATCTGAACCTGTTAAGAAG 71

ACAGAACCAGCCAAAAAGCCAACAGAAGTAGACTACATGAAAGCGAAATCAGTTCACGAATTTACTGTCAA 142

MKAKSVHEFTVK

AGACATCCACGGTAATGAGGTAAAACTCGACAGGTATAAAGGGCAAGTACTTATAATCGTCAATGTGGCCT 213

DIHGNEVKLDRYKGQVLIIVNVAS

CCAATTGCGGCTACACCAACGTACATTACAAACAACTGAACGAACTATACGAAAAGTATAGCAATAAGGGT 284

NCGYTNVHYKQLNELYEKYSNKG

CTACGTATACTGGCCTTCCCTTGCAATCAGTTCGCTTACCAAGAACCAGGGAGCCCTGAAGAAATCCTCAA 355

LRILAFPCNQFAYQEPGSPEEILK

ATTCACTAAGGCTAAACAAGTAAAATTCGATTTGTTTGAGAAGGTCGCGGTCAATGGGGAA 416

FTKAKQVKFDLFEKVAVNGE

***SePOX-C***

TGGACGTGTGTGTTGTTCGTCGTTCTTTACTGTTTCGTCACATTTTACCCATTTCAACAAGATGCCTCTC 70

MPL

CAGCTGACCAAGCCCGCTCCCCAGTTCAAGACCACCGCTGTAGTCAACGGCGAGTTCAAGGACGTCTCTC 140

QLTKPAPQFKTTAVVNGEFKDVSL

TCTCCGACTACAAGGGCAAATATGTAGTACTGTTTTTCTACCCATTGGACTTCACTTTTGTGTGCCCCAC 210

SDYKGKYVVLFFYPLDFTFVCPT

TGAAATCATCGCGTTCTCCGACCGTGCTGATGATTTCCGTAAGATTGGCTGCGAGGTCATTGGTGCCTCC 280

EIIAFSDRADDFRKIGCEVIGAS

ACCGACTCTCACTTCACACATCTCGCCTGGATCAACACCCCACGTAAACAGGGTGGTCTCGGTCCCATGA 350

TDSHFTHLAWINTPRKQGGLGPMN

ACATTCCCCTGATCAGTGACAAGTCTCACCGCATCGCTCGTGATTATGGAGTGTTGAACGAGGAGACTGG 420

IPLISDKSHRIARDYGVLNEETG

CATCCCATTCAGAGGCCTGTTCATCATTGATGACAAGCAGAACCTTAGGCAGATCACCGTCAACGACCTG 490

IPFRGLFIIDDKQNLRQITVNDL

CCTGTTGGCCGATCCGTGGAGGAGACCCTCCGTCTGGTGCAGGCGTTCCAGTACACAGACAAGTACGGAG 560

PVGRSVEETLRLVQAFQYTDKYGE

AGGTGTGCCCCGCCAACTGGAGGCCTGGCGCGAAGACCATCAAGCCCGACACCAAAGCTGCCCAGGAATA 630

VCPANWRPGAKTIKPDTKAAQEY

CTTCGTCGACGCCAACTAATCACTCTCTATACAAAACCACGTAGGCGCGGCTGAACGTTGATATCTCTCG 700

FVDAN

CTAGTTCGAGCACCAACCAAAAGATGCCGCTTTTATAAAAATCGTATAACTTTTGGTCTTAACATATCGA 770

AATTCAGCCGCAAATAAATTAGTTCCCTTATCAACGAAATTGCACTTATGATCATCATCATCATCATCAG 840

GCCTCTGTTATAGTCGCAAGTGCAATTTTCGCTCTAGCACACTGAATGCCGAAAATTGGAGTTGAGAAGT 910

AATCTGAATCTCAGTGATAGGCCTGTTCATCTTTCTGTCTTAACAACCATCAAATAAATGAAAGGACAAG 980

TATAACAGGTGAAAACTCCTATTTTCCTTTACAGTTGGACAATAATAAAATGTAGGTCACTAAGGTGCTT 1050

AGTACCTCATACGCAATCAGTATTCAACGGAGATCCTATGATATTGTACTCTACAATCGAGGCTGTAATA 1120

AAAGCTATTGTAACTAAAAAAAAAAAAAAAAAA 1153

***SePOX-D***

AACCCGTTTTCTTTCTAGACATACTAATACTATGTTCAATTGTATGCAAAAAGTACACTTATATAAATGCC 71

MQKVHLYKCR

GTAAGGTTCATATTTGTGTGGAAGTATTAGTTGGCTTTTTCGAAGTATTCCTTGCTAGCTTTGGGACTAGG 142

KVHICVEVLVGFFEVFLASFGTR

CTTGATCGTGGCTGCGTTTGTGTCTGGGTTCCAATTGGCTGGGCAGACTTCGCCATGTTTGTCAGCAAACT 213

LDRGCVCVWVPIGWADFAMFVSKL

GGAAGGCCTTTACAAGCCTGAGGGTCTCATCAACAGATCTACCGACAGGCAGGTCGTTGACCGACATATGT 284

EGLYKPEGLINRSTDRQVVDRHMS

CTAAGAATACCGTTTCTGTCAATCACGAACAGACCTCTAAGAGCGAAACCAGCATCTAACAGGACATTGTA 355

KNTVSVNHEQTSKSETSI

GTCTTGTGAAATCTTCTTGGAGTAATCAGCAAGAAGAGGGATTTCTATCTTGCCGAGACCACCATCCTTTC 426

TCGGAGTGTTAGTCCAAGCAAGATGGCTGAACTCAGAGTCAGTGGAAACTCCAATAATCTGGCAATCAATA 497

CTGGCAAACTCCTTTGCCCTGTCACTGAATGCAATCAGTTCAGTCGGGCAGACAAAGGTAAAATCAAGTGG 568

GTAGAAAAATGAGAACAACATATTTTCCATGGTAATCTGCCAGCTTCAATTTGTTGAATTCGCCATTGATC 639

ACTGCAGTTGCTTCAAAATTAGGCGCGGGCTGCTGAACTTTTGGTACAAACACTGCACTAGTTGTAGAAAA 710

GTTAACTTTTTTTACCGATGCTACAGCTGGAGTGAAGACTCTGCGAGCCAGCTGTTTTACCAAGAAAGACA 787

TTTTGTTTATAATTGATTGCT 808

***SePOX-E***

GGAGGATCGTCATAGCTGAAATACAGCATATCACGTACCAAGAGTGGTTGCCTGCTAACTTTGGAGAATAC 71

TACCTCCACTACTACGGGATATCTCCAACAACGCTGTATACTCGAGACTACAATCCCGATGTGAACCCTGG 142

GATCATCAACAGTTTTGGCGCGGCTGCCTTCCGGTTTCTGCACACTGTCATCTCCGATAATATCATGACCT 213

MTC

GTCCTAATAGTTACAATGCGGCTTACTTGTACAAATTAAGTGACCATTACTTCAACCCGAGTCTTCTGGAA 284

PNSYNAAYLYKLSDHYFNPSLLE

TGTTCTCCAGACTCATTTGACGATGTTGTACGAGGGATCATAGCTCAGAATGCAGGGGAATCAGACCCCTA 355

CSPDSFDDVVRGIIAQNAGESDPY

TTGTTCAGGGGAGATCACCAACTTGCTGTTCAAATCCCGTAACCGATGGGGCATGGACCTCATAGCTATGG 426

CSGEITNLLFKSRNRWGMDLIAMD

ACATACAGCGGGGCAGGGACCATGGGATCGCGTCGTATAATGACTTAAGGGAAATCTGCGGTTTACCCAGA 497

IQRGRDHGIASYNDLREICGLPR

GCAAGATGCTTTCAGGACTTAGCGAATGAAATTTCACAAGATCGGATAAACGCACTTCAGTATTTGTACGA 568

ARCFQDLANEISQDRINALQYLYE

ATGCGTGGACGATATAGATTTGTTCGTGGGCGGTGCCATGGAGAGGGACGTGTATGGCTCCATACTTGGCC 639

CVDDIDLFVGGAMERDVYGSILGQ

AAACCTTCCAATGTATAGTGGCCGAACAATTCTACAGGACTCGGATATCTGACCGATACTTCTATGATAAT 710

TFQCIVAEQFYRTRISDRYFYDN

GGGGAGATGCCGCATTCTTTTACAAGTGATCAATTAAAAGAACTGAAGAAGGCATCAATGGCTCGTTTGAT 781

GEMPHSFTSDQLKELKKASMARLI

CTGCGACAATACAGACAGCGTGTACTACGTACAGAAGAAAGCGTTTGAAGTGGAATCTACGTACAATCCAA 852

CDNTDSVYYVQKKAFEVESTYNPK

AGTATAGATGTGATGATTACAATGCTATACCCTATGTGGATCTTACTGCTTGGAAACAACCTTCAATATTT 923

YRCDDYNAIPYVDLTAWKQPSIF

GATTGA 929

D

***SePOX-F***

ATGGCGGGAGTGACAATTTTACTGGCGCTGGCGTTGTGCTGTGCTGCACAGGGCATTCACCTCAACTTCC 70

MAGVTILLALALCCAAQGIHLNFR

GACCGACAACATCGCAACTGTTCTTGCAAACCACACCAAGTCTACAAATCACTGGACCTGGAGCCTCCTT 140

PTTSQLFLQTTPSLQITGPGASF

CGCGAGAGTTGTAGCATTTGCCCCCCAAACTGAAAGACGACGTGTTACAGGATTCGGCCCGACTCCTGAT 210

ARVVAFAPQTERRRVTGFGPTPD

GCAGCCTTCTTCGGACCAGCTCCAAGTTTCAGCAATCCACCTCCTCCTGCTGGCTCGGAACTGGACCCAG 280

AAFFGPAPSFSNPPPPAGSELDPA

CTTCTCAAACCTGTGGACTTGCGCCTCCATTCTGCGCCAAGTCTCGTTACAGAAGTATTGATGGTACTTG 350

SQTCGLAPPFCAKSRYRSIDGTC

CAATAACCTCCAAAGGCCAGATTGGGGTATTTCTAATGCGGCTTTTGGTCGGATAGCGCCTGCAGATTAT 420

NNLQRPDWGISNAAFGRIAPADY

GATGACGGTGTAAGTGCTATAAAGACCCTTTCGAAGACGGGCCGACCTTTGCCGAATGCTCGTGAACTTA 490

DDGVSAIKTLSKTGRPLPNARELS

GTCTACGGCTGTTCCCTGACCTGCATGTCATTGACCCCGTATGGACCCTAAACACCCAACAATGGGGACA 560

LRLFPDLHVIDPVWTLNTQQWGQ

AATTGTGACCCACGACATGTCACTAGCTGCTGGCGAAATACAAGCTCATAAGGAATTGACGAACTGCTGC 630

IVTHDMSLAAGEIQAHKELTNCC

GATGACAACGGAAGACTCACTGATACTGCGCAATCGAATCCATCTTGTGCACCTATACTGATACCTCGTA 700

DDNGRLTDTAQSNPSCAPILIPRN

ATGACCCTGTGCATGCACCACAAGGAACTCAGTGTATGAACTTTGTAAGGACGGGAACGACTAGAGACAG 770

DPVHAPQGTQCMNFVRTGTTRDR

AGGATGCACTCCACCTAACGTCCCTGCTCAACAGCTAACAGCGGTCACAGCCTTTATGGATCTCTCTTTG 840

GCTPPNVPAQQLTAVTAFMDLSL

GTCTACGGTAGCAGTCAGACTCAGGCCGATCCAATCCGAGCCCGTCAAGGAGGTCGTCTTTTGACCATTG 910

VYGSSQTQADPIRARQGGRLLTIV

TCAGAGGAGGCCGGGAATGGCCGCCACAGGAACCTAACACGACCGTCATCTGTGAAACTGCGCAGTCACC 980

RGGREWPPQEPNTTVICETAQSP

TAATGAACCCTGTTATTTGACCGGTGACATTCGTGTGAACCAAAACCCTCAGCTGACGACTCTCCAAGTG 1050

NEPCYLTGDIRVNQNPQLTTLQV

ATACTCATGAGGGAACACAACCGTATTGCTGATACTCTAGCTCAACTCAACCCTCACTGGAATGACGAGA 1120

ILMREHNRIADTLAQLNPHWNDET

CTCTCTTCCAAGAAGCGAGACGTATCCACATAGCTGAGATACAACACATCAATTACTATGAATACCTTCC 1190

LFQEARRIHIAEIQHINYYEYLP

TATTTTGTTAGGTTTTGAGAATATGGTGAAGAACAAGCTCATCTACCCTGGTGCTCACGGCTACGTGAAC 1260

ILLGFENMVKNKLIYPGAHGYVN

GATTACAACCCTGGAGTGGATCCTTCCATACTCAATGAACACGCCACAGCTGCCTTCCGTCATTTCCATA 1330

DYNPGVDPSILNEHATAAFRHFHS

GCCTGATCAGAGGTCACTTGAAATTAATCTCAGAAACCCGTCGCGCTATTGGAGCCGTGCGCATGAGTGA 1400

LIRGHLKLISETRRAIGAVRMSD

CTGGTTTACCCGACCGCTACTGTTGGAACTCGACAATGCCTTCGATCACTTGGTTCGAGGACTTACAACT 1470

WFTRPLLLELDNAFDHLVRGLTT

CAGGAGCAAGACTTCAGTGACCAGTCCTTCGATAGTGAAATTACTCAGTTCCTGTTTAAGCGTAACAATA 1540

QEQDFSDQSFDSEITQFLFKRNNT

CTTTTGGCGGTGATCTGCGTGCAAGAGATATCCAAAGAGGTCGTGACCATGGCTTGGCATCCTATATAAC 1610

FGGDLRARDIQRGRDHGLASYIT

TAGCAGAGCTGCGTGTGGCCTTCCTGTTCCCAAGACTTTCACTGATATGTTGGACTTCATATCTCATGAG 1680

SRAACGLPVPKTFTDMLDFISHE

AACGTGGCTGTCCTTCAAAACTTGTACGCGACTCCTGAAGACGTAGAACTGGTGGTAGCTGGTTCTTTGG 1750

NVAVLQNLYATPEDVELVVAGSLE

AGCGCAACGTGCCAGGAGCTCAAGCTGGTCCAACCTTCCTCTGCATCATGACGGAACAGTTCTACAGAAC 1820

RNVPGAQAGPTFLCIMTEQFYRT

ACGTGTGGGAGACAGATACTTCTATGAAAATGGCGCTGATCCTGACATTGCTTTTACGCCAAGCCAGCTC 1890

RVGDRYFYENGADPDIAFTPSQL

GACACGATACGCAAGGGAGCATCCATGGCGCGATTGCTATGTGATAACAGTGACGGAATACAGGCTATGC 1960

DTIRKGASMARLLCDNSDGIQAMQ

AGCCCAGAGCCTTCCAACAGATATCGCACACGAATGTGCTGGTACCCTGTCAATCATTACCAGCTATTGA 2030

PRAFQQISHTNVLVPCQSLPAID

TCTAACTCTATGGCAAGACGCAAGAAGCCATTTTTAA 2067

LTLWQDARSHF

***SePOX-G***

ATGGAGTACACCCGATCCGTCACTACCTACAGAGGAGATTGCACTTTCGGAGCTGCTGAGCAGATGAAT 70

MN

CAAGCTACCCATTTCCTCGATGGATCAAACATCTATGGCGCAAATAGCTATGATGCGGCAGCCCTACGT 140

QATHFLDGSNIYGANSYDAAALR

GAAAGAACCGGGGGACTTCTAAAGACTGCTCAAATTGAAGACGAGGAACATCTGCCATTAGCTGCCAAC 210

ERTGGLLKTAQIEDEEHLPLAAN

CCTACTGAACAGTGCTTAGTGGACAGCAAAACTGGAACTTGCTTTAACGCCGGTGATGCCCGTGCCAAC 280

PTEQCLVDSKTGTCFNAGDARAN

ACCCACCCATGGCTAGCGAGTATGTACTCCATTTGGGTGAGAGAACACAACCGCATTGCCCGTACCCTG 350

THPWLASMYSIWVREHNRIARTL

GCCACTCTCAATCCTGGCTGGAACTCCGACCGTCTGTACCATGAAGCTAGAAGGATTGTCATTGCTGAA 420

ATLNPGWNSDRLYHEARRIVIAE

CTCCAACACATAACCTACAAGACCTGGTTGCCTGCACTTACCGGAAGGTCATTCGATGAGCTATATGAG 490

LQHITYKTWLPALTGRSFDELYE

AGTTATGACCCAGGATACGTCTCGGAGATTGACCCCACAATCACGAACTCGTTTGCCACCGCCGCCTTC 560

SYDPGYVSEIDPTITNSFATAAF

CACTTTGTCAATAGCCTTCTCGACCAGGACATTGAACTCGTTGACAAAGACAACAGTGTGACGCCACAC 630

HFVNSLLDQDIELVDKDNSVTPH

CGTCTTCTAAACAACTACTTCAAACCAGAGCTGGTCTCACAAAAGGGAGGGCTTGAGAAGATCTTGAGG 700

RLLNNYFKPELVSQKGGLEKILR

GGAATGGTTAGCCAGAAGAGCCAGGGATTGGACTTCAATTATGATGATGACCTCCGTCACCAATGGCTG 770

GMVSQKSQGLDFNYDDDLRHQWL

GGTGGTCTTGATGTGTTGGCTGTGGACATCCAACGCGGTCGTGACCATGGTCTACCTGGATACACTCAG 840

GGLDVLAVDIQRGRDHGLPGYTQ

TACCGCACCCTCTGTGGCTTGCCTGCCGTCTCCAGCTTCCAACAACTTAGTGACGTTATGCCAGAAGAG 910

YRTLCGLPAVSSFQQLSDVMPEE

ACAATTACAAAGCTATCGAAACTGTACGAGCAGCCTGGTGATATCGACCTGGTCATTGGTTTGATGGCT 980

TITKLSKLYEQPGDIDLVIGLMA

GAGTCACCGGTCCCTGGATCTCTTTTGGGACCTACAGCCATTTGCATCATCAAGGAACAACTTTGGCGC 1050

ESPVPGSLLGPTAICIIKEQLWR

ACAAAGGCAGGAGACAGATACTTCTACAGTCACCAAGATGAAGCCGGCAAGTTCACCAAGAGACAGCTT 1120

TKAGDRYFYSHQDEAGKFTKRQL

GCTGAAATAAAGCGCTCATCCCTCGCAAGACTACTTTGTGACAACACGCCTTTCGATAGAATTCAGAAA 1190

AEIKRSSLARLLCDNTPFDRIQK

GATGCCTTCCAACCGGTTTCTGAAAGCAATCCTATCGTCCCTTGTGAAGAAATCAAAAAGGTCAACTTG 1260

DAFQPVSESNPIVPCEEIKKVNL

GAAGCGTGGCAGGCCTCAACAGAACAACCAGACATTCTAACTCGCACCAACAATTGGCTCAAAAACAAA 1330

EAWQASTEQPDILTRTNNWLKNK

GTCGGCAGCTCGGGAAACTCCACGAAGTAG 1360

VGSSGNSTK

***SePOX-H***

ATGATCGGAGGTGCTATGGAGAAGCCAGCGTCTGGTGCTGTAGTTGGATCCACCATTGCTTGCGTTTTAG 70

MIGGAMEKPASGAVVGSTIACVLA

CTCTGCAATTCGCCAACTTGAAAAAGAGTGACAGATTTTGGTATGAAAATGACTTGCCCCCATCATCGCT 140

LQFANLKKSDRFWYENDLPPSSL

GTCACCCGATCAGCTTGGTGCCATCAGAAAAGTATCTCTCGCTGGTACCCTCTGTGCTGCTCAAGATTAT 210

SPDQLGAIRKVSLAGTLCAAQDY

CTATCAAACATTCAACCAAAAGCTTTCGTGAGGGAGGATCCTTACCTCAACGCAGCCCAACATTGCTCAC 280

LSNIQPKAFVREDPYLNAAQHCSQ

AGCACAACCGTCTAGAACTTTCTGCGTGGAGAGATGAAAGCGGTGCCAAGGCCGCGGAGAGATTGTCACA 350

HNRLELSAWRDESGAKAAERLSQ

GGACATGCTCGCTACTGCTCTAGAGAAAGCTAAACAAGAGATGGCTGATAGGAAGAAACTCGAATATATG 420

DMLATALEKAKQEMADRKKLEYM

TTGTGGGAAGCACGTGGAGGAGCCGATCCCAAATCTCCAGTTGGTACAGCAGCTTCATTCTCCAAGGCTA 490

LWEARGGADPKSPVGTAASFSKAN

ACAAATATGCTCTGAAACTGGCCAACACATCACTGTTCCTCGAGTTCGCTACCAATGAACTTATCAACAC 560

KYALKLANTSLFLEFATNELINT

TATCGGCACCAACCATCGTCGCAAGCGTCAGATCTTTGACGACTCTCTCGGTTTTGGCACCACGGACTTT 630

IGTNHRRKRQIFDDSLGFGTTDF

GTAGACTCTTTACAATCGGTTGACATCAGCGGTTTTCTAGGCAATGACCAGTCAGGGCCCATCATCGAGC 700

VDSLQSVDISGFLGNDQSGPIIEP

CCCAATGTGATGACAAGGGATCATGCGAACCCGACAATCCTTTCAGAACATACACAGGATACTGTAACAA 770

QCDDKGSCEPDNPERTYTGYCNN

TTTGAGAAATCCTAACTTGGGCAAGGGATTGACTACTTTCGCTAGGTTGCTACCTCCTGTTTATGAAGAT 840

LRNPNLGKGLTTFARLLPPVYED

GGTGTAAGTCGTCCCCGCATCAACTCGGTCACAGGCACGCCCCTACCATCTCCTCGTGTGGTATCTACAG 910

GVSRPRINSVTGTPLPSPRVVSTV

TAATTCACCCCGATATCTCCAATCTCCATACACGATACACCCTCATGACTATGCAGTTCGCTCAGTTCCT 980

IHPDISNLHTRYTLMTMQFAQFL

TGACCATGAACTTACTATGACGCCCATCCACAAAGGTTTCCACGAGTCTATCCCCGACTGTCGGTCCTGT 1050

DHELTMTPIHKGFHESIPDCRSC

GACTCTCCTCGCACGGTGCATCCTGAGTGCAATCCGTTCCCAGTACCACGTGGAGACCACTACTACCCAG 1120

DSPRTVHPECNPFPVPRGDHYYPE

AAGTGAATGTTACTTCTGGAGAGAGGCTCTGCTTCCCCTTCATGAGAAGTTTGCCTGGTCAACAGCAACT 1190

VNVTSGERLCFPFMRSLPGQQQL

TGGTCCTCGTGAACAAGTAAACCAGAACACAGCCTTCATCGACGCCTCAGTGATCTACGGTGAGAATCCT 1260

GPREQVNQNTAFIDASVIYGENP

TGCATCGTACGCAAATTGCGTGGTTTCAACGGAAGACTGAACGCTACCACCAACCCTGCAAATGGCAGAG 1330

CIVRKLRGFNGRLNATTNPANGRE

AATTGCTGCCTAGAAGTGACAGCCATCCTGAGTGCAAAGCTCCCAGCGGATTTTGCTTTATTGCTGGTGA 1400

LLPRSDSHPECKAPSGFCFIAGD

CGGACGAGCATCAGAACAGCCAGGTTTAACCGCAATCCATACAATCTTCCTTCGCGAGCACAACCGCATC 1470

GRASEQPGLTAIHTIFLREHNRI

GTGGAAGGTCTTCGCGGCGTCAACCCGCACTGGGACGCCGACCAACTGTTCGAACACACTCGCCGCATCG 1540

VEGLRGVNPHWDADQLFEHTRRIV

TCGCCGCCACCTTCACGCACGTCATCTACAACGAGTTCTTGCCAAGATTGCTCTCGTGGAACGCTGTCAA 1610

AATFTHVIYNEFLPRLLSWNAVN

CTTGTACGGACTCAAATTGCTGCCTTCAGGCTACTACAAGGAATACTCCCCGACCTGCAACCCTGCCATT 1680

LYGLKLLPSGYYKEYSPTCNPAI

GTCACTGAGTTCGCGACAGCTGCCTTCCGATTCGGTCACTCACTGTTACGCCCTCACCTTCCTCGACTGT 1750

VTEFATAAFRFGHSLLRPHLPRLS

CGCCCAGCTTCCAGCCCGTGGAACCACCAATCCTACTCCGAGATGGATTCTTCAGGCCTGATATGTTCAT 1820

PSFQPVEPPILLRDGFFRPDMFM

GTCTCATCCACCAATGGTAGACGAACTGATGCGTGGTCTAGCTTCCACTCCCATGGAAACCCTCGACCAG 1890

SHPPMVDELMRGLASTPMETLDQ

TTTATCACTGGTGAAGTCACCAACCATCTCTTTGAGGACCGCCGTATTCCCTTCTCTGGTGTCGATCTGA 1960

FITGEVTNHLFEDRRIPFSGVDLI TTGCCCTGAACATCCAAAGATCAAGAGACCACGGTATCCCGAGCTACAACAACTACAGAGCACTTTGCAA 2030

ALNIQRSRDHGIPSYNNYRALCN

TTTGAAGAGAGCGACTACCTTCGAAGATTTGGCTAGAGAAATTCCCGATGAAGTTATTGCTAGGTTCAAG 2100

LKRATTFEDLAREIPDEVIARFK

CGCATCTACGCTACAGTCGACGACATCGATCTATTCCCTGGTGGTATGAGCGAGAGACCGCTGCAGGGAG 2170

RIYATVDDIDLFPGGMSERPLQGG

GTCTGGTTGGACCTACCTTCGCCTGCATCATCGCCATTCAGTTCAGACAGCTTAGGAAATGCGACAGATA 2240

LVGPTFACIIAIQFRQLRKCDRY

TTGGTATGAGAACGACAAC 2259

WYENDN

***SePOX-I***

GTTGTTGTGGTTTTCCTAAGATGTTTTTTAGTAGTCTCTTCAGATTTGCTTATATCATTATCAGTTTCAG 70

CATCCTCATCTATTTCGACCCAGGGAGATAAGTCAAAGTTATTGAGTAGTCCGTTCTGGCAGGATATCCT 140

GTCATTGTCAGGGTTGTCAGTTGAAAGGAAAACGAAAGGCTGTACAGTGTCTATGCTGTCTAATGTACGG 210

CAGAGAATTTGCGCGAAAGAGATCCTACGAATTTGTTGAAGTTGTGCTGGCGTAAATGATGATTCAAAGC 280

CACCATTTTCGTACCAAAATCGATCTCCTTTTCTGAGATTGCTGAACTGCTGTGCTATGATGCAGGCGAA 350

MQAN

CGTTGGTCCCACAAGACCACCAACTACTGGTCTCTCTGCCATACCCGCGGTGAATAGATCAATATCGTCT 420

VGPTRPPTTGLSAIPAVNRSISS

ACGTGCCTGTACAGTGCCTTCATCTTCCTCGCAGCCCTTGCTGGCATTACCCTAAGCAGGTCTTCAAAGT 490

TCLYSAFIFLAALAGITLSRSSKS

CTTCAATAGTAGACAACCCACAAGGTTCTCTCCATGATGTATACGGAGGAACTCCATGGTCCCTCCCCCT 560

SIVDNPQGSLHDVYGGTPWSLPL

CTGAATGTTAATAGCCGCGAGGTCCATTCCAAAGTCGAAATGAGGAGTTTGGAAAAGATGGTTGGTGAGT 630

TCTCCCGTGATGAACTCATCGCGTTTCTGTATCGGCTGGTTCAGCATACCGAGGAGAAGACGGTCTACTG 700

CTCCGATAGACCAGATGTTCGAGGGGTTTGTTAGTTCGTCGTGGAGAGAAACATTGTTACTCATAGGTCT 770

GTGGAACCTATCGAATCTAACCATGGAAGACTGCACGAGGCTGTGGCCGAACCTGAAGGCTGCTGAGCCA 840

AACGAGCTAGCTGGACTAGGGTTGATCTTAGGGTTGTAACCCTTAAAGTACCCTTTCTTCTGCAGTTCCA 910

GCTCAAAGCGATGCATCACCTCAGAACCCAGCACAATAGGCAGGAACTCTCTGTAAGTAATGTGCTGTAT 980

CATGGCTCCCAAATGTCCTCCTGCTTCTAAAAGATACCGCACCCACGATGGCACATGCAACAACATCAAC 1050

CGTCCCCGATGGGGAGCCACCATGA 1075

***SePOX-J***

ATGGACAAGTTGATAATTTTATTTCAGTTTGTGTTTATTTGTTCCGTTTTGGGAGATGCGACATATTATA 70

MDKLIILFQFVFICSVLGDATYYS

GTTCCTACTATGGCTTACCTTTGACCGCTTCTCAAGTTGCTGCTTACACATCAAATGGCACCGTTGCAAA 140

SYYGLPLTASQVAAYTSNGTVAN

CTGTACAATTTCGGTGGAGGCCTGCGATGAAACTGAAGCTAGGCGTATTGATGGAACATGTAACAACCTG 210

CTISVEACDETEARRIDGTCNNL

AACTACCCGTCATACGGTGGTACTCGCACTCCTTACTACAGAATCCTCGATGCATCTTACCACAAGAAAT 280

NYPSYGGTRTPYYRILDASYHKKS

CAAGTTCAGAATTTGAGCCAAGGCTTTCGGCCTCGGGAACTGAACTAAACCTCACAAGGAAAATCAGAAC 350

SSEFEPRLSASGTELNLTRKIRT

TTCAATCTGGGCTGAAGGACGAGTGGACGATGAGGTACTTACCAGCGTTATCAACCACATGGCTGTTTTC 420

SIWAEGRVDDEVLTSVINHMAVF

TTCGCTACAGACATCACCAACACTCGTGACACAACAAACTACGTGACTTGGAGACCATACTGCTGCAAGA 490

FATDITNTRDTTNYVTWRPYCCKS

GTGCAGGGAAAAATGACTACGCTTGCACGCCTAACCATGTGCCTAAAGATGACTTCGTGCACCGATTCTC 560

AGKNDYACTPNHVPKDDFVHRFS

TGGAATCAGATGCTTGAACATGACCAGACCTCTGACCTTCCAGACTTCTGGATGTGCTCCCAACACAACT 630

GIRCLNMTRPLTFQTSGCAPNTT

ACACCATTGAGAATCGTCGATGCTACTCCCCTGGTAGACTTGTCCCCTGTTTACGGTAACACCATTGAAA 700

TPLRIVDATPLVDLSPVYGNTIEK

AGGCAAGACGCACAAACTCAGGCGGAAAGCTCGTCATTGTAACAGTCAATGACAGAATTAATTTCCCAGA 770

ARRTNSGGKLVIVTVNDRINFPD

CACATCAAGCCTAAATCTTCTTCTTGGAATCAACTTTGTGGGTATTATTCTCTTCTGGAACAACCACAAC 840

TSSLNLLLGINFVGIILFWNNHN

TTTATCGCCGACAAGCTTGCTGAGGTCAATCCCTGCTGGACAGATGACCAGCTCTTTGACACGGCTAGGG 910

FIADKLAEVNPCWTDDQLFDTARE

AAATTAACATCGCTTATGGTGTTCAAATGTTCTACTATGAACTTATGGCTACTTTGATGGGAAAGGAAAA 980

INIAYGVQMFYYELMATLMGKEN

CTTGATCGCCGATGGTATTTTATCGGAAGATGAAGGCTTCAGGGATCTATATGACGATACCAAACTGCCA 1050

LIADGILSEDEGFRDLYDDTKLP

CAAATTGCTTTGGAGTATCCAGTAGTTTTGAGATGGATGCATTCTCTACAAGACGGTGACTTAAGAATGT 1120

QIALEYPVVLRWMHSLQDGDLRMY

ACTACGCTAACGGAACATACAAATCTACCTTCCCCATCGTAGACCTCACCTCAAACACTGACTTCCTGAA 1190

YANGTYKSTFPIVDLTSNTDFLK

GGTTAACGACACCATGGACTACGTGACTCAAGGGTTCTTTAGACAGCCCACAGCCAATTCTAATGATTAC 1260

VNDTMDYVTQGFFRQPTANSNDY

ATTGCTGATCCTGATATTGTTCAAAGAGGTCTTGGTCGGCTCCAGAGGTCTAATGACATTATGACAAATG 1330

IADPDIVQRGLGRLQRSNDIMTND

ATTTGGCGAAGAACCGTTACTTTGGTTTCAAAGGATACACTGAGTACAGGAACCACTGCTTCGGTGATTC 1400

LAKNRYFGFKGYTEYRNHCFGDS

CATTACCAGCTTCGATGACTTAAGTGATATCATTGATGTTGAGAGGGTAACGCAGCTGAAACAATTGTAC 1470

ITSFDDLSDIIDVERVTQLKQLY

AAAGACGTCACTGACATCGACCTACTTGCCGGGATATGGGTGGAGAAGAAGAAAGAGAACTCACATGTAC 1540

KDVTDIDLLAGIWVEKKKENSHVP

CTCCCACCTTCTACTGTCTCGTCAAGGAACAGTTGGAAAGATCAATAGTCTCAGACAGACATTGGTATGA 1610

PTFYCLVKEQLERSIVSDRHWYE

AAGAGAATCAAGGCCAAATGCTTTCACTGCTGATCAATTAGCGCAAATCCGCAAAGTAAGTTTGGCGCGT 1680

RESRPNAFTADQLAQIRKVSLAR

ATGCTGTGTGATATCGGACCCGGTGTCACTGAAATCCAGCCCAATGCATACGAGTTACCTACGACTGGAA 1750

MLCDIGPGVTEIQPNAYELPTTGN

ACGAGATCGTCAGTTGTGATGAAATTCCCAACTTGGACTACTCATACTGGGCGGATAATACAAAATGCAC 1820

EIVSCDEIPNLDYSYWADNTKCT

TACATCATCATAG 1833

TSS
